# Low Serum Hepcidin in Patients with Autoimmune Liver Diseases

**DOI:** 10.1371/journal.pone.0135486

**Published:** 2015-08-13

**Authors:** Aggeliki Lyberopoulou, Georgia Chachami, Nikolaos K. Gatselis, Eleni Kyratzopoulou, Asterios Saitis, Stella Gabeta, Petros Eliades, Efrosini Paraskeva, Kalliopi Zachou, George K. Koukoulis, Avgi Mamalaki, George N. Dalekos, George Simos

**Affiliations:** 1 Laboratory of Biochemistry, Faculty of Medicine, University of Thessaly, Larissa, Greece; 2 Department of Medicine & Research Laboratory of Internal Medicine, Faculty of Medicine, University of Thessaly, Larissa, Greece; 3 Laboratory of Molecular Biology and Immunobiotechnology, Hellenic Pasteur Institute, Athens, Greece; 4 Laboratory of Physiology, Faculty of Medicine, University of Thessaly, Larissa, Greece; 5 Department of Pathology, Faculty of Medicine, University of Thessaly, Larissa, Greece; 6 Institute for Research & Technology—Thessaly (IRETETH), Larissa, Greece; RWTH Aachen, GERMANY

## Abstract

Hepcidin, a liver hormone, is important for both innate immunity and iron metabolism regulation. As dysfunction of the hepcidin pathway may contribute to liver pathology, we analysed liver hepcidin mRNA and serum hepcidin in patients with chronic liver diseases. Hepcidin mRNA levels were determined in liver biopsies obtained from 126 patients with HCV (n = 21), HBV (n = 23), autoimmune cholestatic disease (primary biliary cirrhosis and primary sclerosing cholangitis; PBC/PSC; n = 34), autoimmune hepatitis (AIH; n = 16) and non-alcoholic fatty liver disease (NAFLD; n = 32). Sera sampled on the biopsy day from the same patients were investigated for serum hepcidin levels. Hepatic hepcidin mRNA levels correlated positively with ferritin and negatively with serum γ-GT levels. However, no correlation was found between serum hepcidin and either ferritin or liver hepcidin mRNA. Both serum hepcidin and the serum hepcidin/ferritin ratio were significantly lower in AIH and PBC/PSC patients’ sera compared to HBV, HCV or NAFLD (P<0.001 for each comparison) and correlated negatively with serum ALP levels. PBC/PSC and AIH patients maintained low serum hepcidin during the course of their two-year long treatment. In summary, parallel determination of liver hepcidin mRNA and serum hepcidin in patients with chronic liver diseases shows that circulating hepcidin and its respective ratio to ferritin are significantly diminished in patients with autoimmune liver diseases. These novel findings, once confirmed by follow-up studies involving bigger size and better-matched disease subgroups, should be taken into consideration during diagnosis and treatment of autoimmune liver diseases.

## Introduction

Hepcidin is a hormone predominantly synthesized and secreted by hepatocytes. It was first identified in human blood and urine as an antimicrobial peptide of innate immunity, named LEAP-1 (liver-expressed antimicrobial peptide-1) and involved in host defense [1, 2]. Soon after, experiments in transgenic mice linked hepcidin with iron disorders and revealed its crucial role in systemic iron homeostasis [3]. Hepcidin binds to ferroportin (FPN), the main iron exporter in mammalian cells and promotes its internalization and subsequent degradation [4, 5]. Under iron-replete conditions, high levels of hepcidin cause iron retention in iron storage cells such as hepatocytes, enterocytes and macrophages and, subsequently, diminished duodenal absorption and release of iron in the bloodstream. In contrast, when there is demand for iron, e.g. when hemopoiesis is stimulated, hepcidin synthesis declines and absorbed or stored iron enters the circulation [6, 7]. Abnormally high serum hepcidin reduces iron plasma concentration and can cause anemia of chronic disease whereas hepcidin deficiency leads to iron overload as in hereditary hemochromatosis [8, 9]. Hemochromatosis can be caused by mutations in the hepcidin gene or in the genes of its regulators hemojuvelin (HJV) and hemochromatosis protein (HFE) [10, 11].

Hepcidin production can be regulated at the transcriptional level by different signaling pathways. Up-regulation of hepcidin under iron-replete hepatic conditions involves the BMP/SMAD-signaling pathway [6, 12, 13] while its up-regulation by elevated transferin-bound iron additionally involves HFE, transferin receptor 2 (Tfr2) and HJV [14–16]. Inflammation and infection can also stimulate hepcidin synthesis by hepatocytes through interleukin-6 (IL-6) and activation of the STAT-3 signaling pathway [17]. In contrast, anemia and hypoxia, both of which necessitate erythropoiesis, suppress hepcidin production by inhibiting the pathways mentioned above or by other poorly characterized mechanisms [6, 18].

Although there are many studies demonstrating altered hepcidin levels in iron disorders, data on hepcidin regulation in chronic liver diseases and in particular autoimmune liver diseases are scarce. Chronic liver diseases can lead to disorders in iron homeostasis [19, 20] and are, especially at late cirrhotic stages, associated with hepatic iron overload, which can contribute to liver injury [21, 22]. Although, deregulation of iron metabolism and distribution can be a consequence of acquired disease symptoms such as inflammation, hepatocyte necrosis or blood loss, liver siderosis can be influenced by genetic factors including *HFE* mutations [23]. As HFE is now known to play an important role in the regulation of hepcidin, alterations in the expression levels of hepcidin may be important for the pathophysiology of chronic liver diseases.

In older studies, liver hepcidin mRNA levels in biopsies were considered as an indicator of hepcidin expression while more recent ones analyze circulating serum hepcidin levels. In both types of studies the hepcidin/ferritin ratio is often used as a variable in combination with patients data. Ferritin, a marker of iron stores, and serum hepcidin correlate positively [24] and as iron load triggers both ferritin and hepcidin production, the hepcidin/ferritin ratio remains stable in individuals with normal liver function but alters when liver dysfunction causes deregulation of the iron-hepcidin axis. The reliability of the hepcidin/ferritin ratio may be questionable because serum ferritin levels can be influenced by factors unrelated to body iron stores, under certain circumstances such as hepatocellular necrosis and inflammation [25, 26]. Nevertheless, a study of patients with chronic liver diseases demonstrated a reduction of the hepcidin/ferritin ratio in relation to increasing fibrosis, suggesting that this ratio may be useful as a potential non-invasive diagnostic marker of cirrhosis [27]. In patients with chronic hepatitis C virus (HCV) infection, who often demonstrate increased hepatic iron load, the liver hepcidin mRNA/serum ferritin ratio was lower compared to healthy controls or patients with hepatitis B virus (HBV) infection [28]. Similarly, the serum hepcidin/serum ferritin ratio was relatively lower in HCV patients compared to HBV [27].

In this work, we evaluated liver hepcidin gene expression, serum hepcidin levels and hepcidin/ferritin ratios in patients with diverse liver diseases, including for the first time patients with autoimmune liver disorders, and subsequently correlated these measurements with the clinical, histological and laboratory data of the patients. Our aim was also to investigate whether alterations of hepcidin expression in liver tissue or in the circulation can be observed and compared between groups of patients at the onset of chronic liver diseases of different etiology.

## Materials and Methods

### Patients and samples

One hundred and twenty-six consecutive patients with chronic liver diseases [52 males, 74 females; age (mean±SD) 46.8±15.5 years] who attended and followed at the outpatient Hepatology clinic of the Department of Medicine, Larissa Medical School, University of Thessaly, Larissa, Greece were included in the study. Disease etiology included HCV (n = 21), HBV (n = 23), AIH (n = 16), PBC/PSC (n = 34; PBC n = 30, PSC n = 4) and NAFLD (n = 32). The demographic, clinical, laboratory and histological characteristics of the patients are shown in Table 1. The sera of seventeen volunteer blood donors [10 males, 7 females; age (mean ± SD) 45.18 ± 15 years] collected at first blood donation were used for serum hepcidin analysis as healthy controls.

**Table 1:** Demographic, clinical, laboratory and histological data as well as hepatic hepcidin mRNA and serum hepcidin levels of patients and healthy controls.

Variables	Total patients (n = 126)	HCV (n = 21)	HBV (n = 23)	AIH (n = 16)	PBC/PSC (n = 34)	NAFLD (n = 32)	Healthy (n = 17)	*P*-value
Age (years)	46.8±15.5	40.6±13.6	42.6±14.6	53.5±16.3	53±13.5	43.9±16.1	45.2±15	0.009
Sex, male / female	52 / 74	10 / 11	15 / 8	4 / 12	7 / 27	16 / 16	10 / 7	0.006
ALT (IU/L)	60 (73)	49 (59)	71 (97)	95 (241)	32 (57)	65 (56)	32 (5)	<0.001
AST (IU/L)	35 (34)	32 (27)	45 (60)	58 (83)	31 (20)	30 (21)	31 (4)	0.027
γGT (IU/L)	45 (69)	27 (34)	28 (50)	30 (88)	71 (77)	57 (66)	29 (8)	<0.001
ALP (IU/L)	76 (42)	63 (30)	68 (41)	71 (39)	105 (100)	81 (34)	62 (12)	<0.001
Bilirubin (mg/dL)	0.66 (0.51)	0.72 (0.35)	0.95 (0.66)	0.65 (1.32)	0.66 (0.48)	0.55 (0.32)	0.65 (0.15)	0.008
Hemoglobin (g/L)	13.8 (2.5)	14.2 (2.3)	14.3 (2.8)	13.4 (1.6)	13 (1.6)	14.5 (2.3)	14.7 (1)	0.003
Ferritin (ng/ml)	119 (124)	91 (114)	143 (207)	126 (213)	88 (120)	123 (99)	NA	0.109
Albumin (g/L)	4.4±0.4	4.5±0.3	4.4±0.3	4.2±0.4	4.3±0.4	4.6±0.3	4.5±0.2	0.001
INR	0.96±0.11	0.96±0.1	0.99±0.12	1±0.12	0.95±0.13	0.92±0.09	0.92±0.1	0.065
Inflammation								0.05
*Minimal/mild (%)*	89 (70.6)	12 (57.1)	14 (60.8)	9 (56.3)	26 (76.5)	28 (87.5)	NA	
*Moderate/severe (%)*	37 (29.4)	9 (42.9)	9 (39.2)	7 (43.8)	8 (23.5)	4 (12.5)	NA	
Fibrosis								<0.001
*None/mild/moderate (%)*	101(80.2)	15 (71.4)	13 (56.5)	11 (68.8)	31 (91.1)	31 (96.8)	NA	
*Severe/cirrhosis (%)*	25 (19.8)	6 (28.6)	10 (43.5)	5 (31.2)	3 (8.9)	1 (3.2)	NA	
Cirrhosis								0.439
*No (%)*	120 (95.2)	19 (90.5)	21 (91.3)	15 (93.7)	33 (97.1)	32 (100)	NA	
*Yes (%)*	6 (4.8)	2 (9.5)	2 (8.7)	1 (6.3)	1 (2.9)	0 (0)	NA	
Liver hepcidin mRNA	0.74 (1.22)	0.72 (1.21)	1.32 (1.08)	0.47 (1.10)	0.74 (1.12)	0.65 (1.18)	NA	0.267
hepcidin mRNA / log ferritin	0.35 (0.61)	0.44 (0.54)	0.61 (0.47)	0.26 (0.53)	0.35 (0.64)	0.29 (0.65)	NA	0.315
Serum hepcidin (ng/ml)	35 (100)	56 (101)	109 (165)	9 (6)	8 (14)	85 (116)	61 (39)	<0.001
Serum hepcidin/ log ferritin	16.9 (50)	26.4 (49.7)	45.9 (72.1)	4.3 (2.9)	4.5 (6.9)	39.4 (60.1)	NA	<0.001

The diagnosis of HCV and HBV was based on the EASL criteria [29, 30]. AIH diagnosis was based on the reported simplified criteria of the IAIHG [31]. According to internationally accepted criteria [32] and previous publications from our group [33–35], patients with PBC met at least two of the following criteria: (1) positivity for AMA; positive titre (≥1/40) either by IIF on in-house rodent tissue substrates confirmed by Western blot using in-house mitochondrial subfraction of rat livers, or by enhanced performance M2 ELISA [M2 EP (MIT3) ELISA, Quanta Lite, INOVA Diagnostics, San Diego, CA], which was shown to have higher sensitivity compared to the conventional anti-M2 [34, 35]; (2) elevated cholestatic enzymes and (3) histological lesions of PBC when a liver biopsy was available. The diagnosis of PSC was based on biochemical or clinical signs of cholestasis, compatible liver histology, repeatedly AMA negativity by IIF, Western blot or ELISA and/or typical findings on endoscopic retrograde cholangiopancreatography or magnetic resonance cholangiography [36]. NAFLD diagnosis was based on the EASL position statement [37]. Six out of the 126 patients were cirrhotic (2 HCV, 2 HBV, 1 AIH, 1 PBC/PSC) but all of them had compensated cirrhosis with preserved liver function. All of them also underwent a gastroscopy, which excluded portal hypertension gastropathy and varices. All patients underwent a thorough investigation in order to exclude other causes of liver abnormalities, including genetic diseases related to iron overload such as hemochromatosis and ferroportin disease. Patients with genetic background that favors iron overload (such as heterozygous hemochromatosis) were not included in the study.

Fine needle liver biopsy was performed for diagnostic purposes. Whenever the material was sufficient, a small sample of the hepatic specimen was immediately immersed in RNA Later buffer (Qiagen, Hilden, Germany) and incubated overnight at 4°C. Then the tissue was removed from the reagent and stored into cryogenic vials at -80°C until further use. Serum samples were obtained from patients on the day of liver biopsy and were immediately frozen and stored at -80°C until used. The study was approved by the ethical committee of the University of Thessaly Medical School. Verbal informed consent was obtained from all participants in the study instead of written consent because the data were analyzed anonymously. The method of obtaining verbal consent was approved by the ethical committee. More specifically, the routine for every patient followed-up in our outpatient Hepatology clinic includes requesting the informed oral consent of the patients for the anonymous use of their samples for potential future retrospective research studies, always provided that there is sufficient sample (e.g. serum, tissue) for definite diagnosis. For this particular study, the required elements of consent were presented orally to the subjects at the day of the liver biopsy, which was always performed for diagnostic purposes. Sufficient time was provided to explain all pertinent information and every patient was free to ask questions. A patient’s relative always witnessed the process. All the procedure of obtaining verbal consent was documented in the source data file of each patient and signed by the treating physician. The procedures followed were in accordance with the Helsinki Declaration of 1975, as revised in 2000.

### RNA isolation

Total RNA from liver tissues was isolated using TRIZOL Reagent (Invitrogen, Carlsbad, CA, USA) along with 10μg RNase-free glucogen (Invitrogen), as carrier to the aqueous phase, according to the manufacturer’s instructions.

### Reverse transcription and quantitative real-time PCR

One microgram of total RNA was reversed transcribed using random primers and 50 Units of Multiscribe Reverse Transcriptase according to the cDNA reverse transcription protocol (Applied Biosystems, Foster City, CA, USA) in a 20μl reaction for 10 min at 25°C, 120 min at 37°C and 5 sec at 85°C. qRT-PCR was carried out using SYBR Green I dye (KAPA Biosystems, Wilmington, MA, USA) in a total volume of 20μl containing 0.5 μM specific forward and reverse primers. Amplification and detection were performed in a MiniOpticon Real-Time PCR Detector (Bio-Rad Laboratories Inc., Hercules, CA, USA) system under the following conditions: 1 cycle at 50°C for 2min and 95°C for 10 min and 40 cycles at 95°C for 15 sec and 60°C for 1 min. The mRNA of human *β*-actin was chosen as internal control in each sample and relative quantification on gene expression was based on the comparative *C*_T_ method, in which the amount of the target was given by 2^−(ΔCT target −ΔCT calibrator)^ or 2^−ΔΔCT^. Primer sequences were designed to specially amplify 123 bp of human hepcidin cDNA (forward 5’-CTCTGTTTTCCCACAACAGAC- 3’, reverse 5’-TAGGGGAAGT-GGGTGTCTC- 3’) [17] and 97 bp of human *β*-actin cDNA, (forward 5’-CCAACCGCGAGAAGATGA-3’, reverse 5’-CCAGAGGCGTACAGGGATAG-3’). The validation experiments were carried out in duplicates and each run was completed with a melting curve analysis to confirm the specificity of the amplification and the lack of primer dimers.

### Hepcidin ELISA

Serum hepcidin-25 determination was performed using a validated ELISA, as already previously described [38].

### Liver histology

Liver biopsy specimens were evaluated by a single experienced liver immunopathologist (GKK) using the Knodell histological/activity index score [39]. According to previous publications of our group and for statistical reasons [40, 41], patients were divided in two groups (i) according to inflammation: minimal/mild (0–8) and moderate/severe (9–18) and (ii) according to fibrosis none/mild/moderate (0–2) and severe fibrosis/cirrhosis (3–4).

### Statistical analysis

Kolmogorov-Smirnov test was used to assess the normality of distribution. Normally distributed values are expressed as mean±SD, while non-normally as median (IQR). Data were analysed by x2 test, Fisher’s exact test, ANOVA, Kruskal-Wallis or Mann-Whitney U test, Spearman’s coefficient of correlation (r) and Wilcoxon Signed-Rank test where applicable. Multivariate analysis was performed in the total group of our patients (n = 126) by a stepwise linear regression with hepcidin levels as the dependent variable, while factors found to be significantly associated with serum hepcidin levels at univariate analysis were included as independent variables. We also included ferritin levels in our multivariate model to assess their impact as a confounding factor. Two-sided P-values <0.05 were considered as statistically significant.

## Results

### Comparison of clinical characteristics and hepcidin expression levels between patients with different liver diseases

Clinical characteristics of the 126 patients, with different liver diseases that underwent liver biopsy, are summarized in Table 1. The patients were middle-aged (mean±SD: 46.8 ± 15.5 years) with a predominance of women (58%). Statistical differences were observed in markers of liver injury and dysfunction including ALT, AST, γGT, ALP and serum bilirubin, between the five groups (Table 1). Serum aminotransferase, ALT and AST, levels were significantly higher in the AIH [median (IQR): 95 (241) and 58 (83), respectively] group indicating that these patients are more susceptible to hepatic cell damage. As anticipated, γGT and ALP levels, which indicate cholestasis, were higher in the PBC/PSC patient group. Serum ferritin and INR levels did not differ significantly between groups and median values were within the normal range, indicating similar body iron stores and functional coagulation pathway in patients with different diseases.

Histological analysis was performed on the biopsy specimens of our patients evaluating levels of inflammation (minimal/mild or moderate/severe), the extent of fibrosis (none/mild/moderate or severe/cirrhosis) and appearance of cirrhosis in liver tissue (Table 1). The differences in inflammation grading between the five groups were at the margin of statistical significance (P = 0.05). The highest percentage of patients with moderate to severe inflammation was observed in the HCV and AIH groups (42.9% and 43.8%, respectively). The differences in fibrosis staging were statistically significant between the five groups (P<0.001). Extremely small percentage of patients with moderate to severe fibrosis is observed in the PBC/PSC and NAFLD groups (8.9% and 3.2%, respectively) while the highest percentage is observed in the HBV group (43.5%) followed by the AIH (31.2%) and the HCV (28.6%) groups. These differences reveal that fibrosis staging in the biopsy specimens of our patients is heterogeneous and depends on disease etiology and progression. Nevertheless, only 6 (4.8%) patients out of the total cohort of the 126 had cirrhosis and no statistically significant differences were observed between the five groups as far as cirrhosis is concerned.

**Fig 1:**
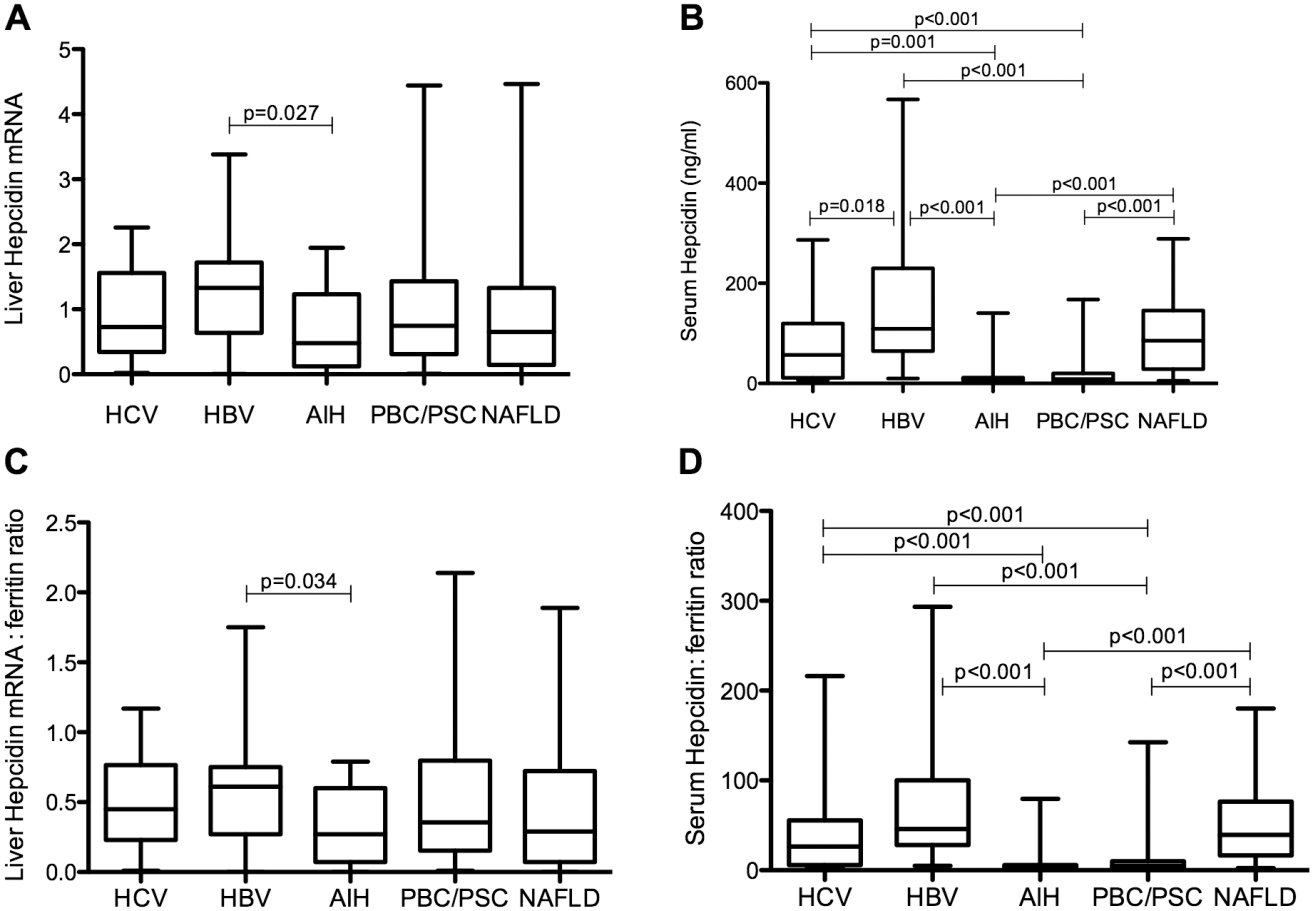
Liver hepcidin mRNA and serum hepcidin levels in various liver diseases. Liver hepcidin mRNA (A), serum hepcidin levels (ng/ml) (B), liver hepcidin mRNA/log ratio (C) and serum hepcidin/log ferritin ratio (D) in the studied groups. Graphs depict the median (line within the box), 25^th^ to 70^th^ percentiles (upper and lower border of the box), and 10^th^ to 90^th^ percentiles (whiskers). *P* values calculated using Kruskal-Wallis t test, following Mann-Whitney U test for comparisons between groups.

Samples of liver biopsies were analysed for hepcidin gene expression by quantifying the levels of hepcidin mRNA using reverse transcription and quantitative real-time PCR. Serum samples drawn on the same day as the biopsy were also analysed using a homemade validated ELISA to quantify the levels of circulating mature hepcidin peptide. Liver hepcidin mRNA, serum ferritin levels and liver hepcidin mRNA/log ferritin ratio did not significantly differ among disease groups (Table 1) with a single exception: liver hepcidin mRNA and liver hepcidin mRNA/log ferritin ratio were significantly different between HBV patients [1.32 (1.08) and 0.61 (0.47), respectively] and AIH patients [0.47 (1.10) and 0.26 (0.53), respectively], being lower in the latter (Fig 1A & 1C). On the other hand, the median serum hepcidin concentration was significantly lower in PBC/PSC [8 (14) ng/ml] and AIH patients [9 (6) ng/ml] compared to HCV [56 (101) ng/ml], HBV [109 (165) ng/ml], and NAFLD patients [85 (116) ng/ml] (Table 1; Fig 1B) or healthy controls [61 (39) ng/ml; P<0.001 for both comparisons]. Importantly, the serum hepcidin/log ferritin ratio was also significantly lower in AIH and PBC/PSC patients compared to the other study groups (Table 1; Fig 1D). Serum hepcidin levels (but not the serum hepcidin/log ferritin ratio) were significantly lower in HCV compared to HBV patients (Table 1; Fig 1B).

### Correlations between hepcidin and demographic, clinical, laboratory and histological characteristics in all patients

Serum hepcidin levels and serum hepcidin/log ferritin ratio negatively correlated with age (Table 2; P = 0.011 and P = 0.009), suggesting lower circulating hepcidin with increasing age. Both median liver hepcidin mRNA and serum hepcidin levels were significantly lower in female [0.57 (1.12) and 20 (93), respectively] compared to male patients [1.01 (1.25) and 57 (126), respectively; P<0.05 and P = 0.02, respectively]. Median serum hepcidin was also lower in healthy females [41 (40) ng/ml] than healthy males [75 (31) ng/ml; P = 0.003]. Lower iron stores can explain the lower hepcidin levels in female patients since there is a strong negative correlation of ferritin with the female gender. Indeed, the liver hepcidin mRNA/log ferritin and serum hepcidin/log ferritin ratios had no statistically significant correlation with gender among the patients.

**Table 2:** Correlations between liver hepcidin mRNA, serum hepcidin, ferritin and clinical characteristics of patients.

Variables	Liver hepcidinmRNA		Serum hepcidin(ng/ml)		Ferritin(ng/ml)		Hepcidin mRNA/log ferritin		Serum hepcidin/log ferritin	
Statistics	**r**	***P***	**r**	***P***	**r**	***P***	**r**	***P***	**r**	***P***
Age	0.11	0.202	-0.23	0.011	0.05	0.598	0.12	0.185	-0.23	0.009
ALT (IU/L)	-0.09	0.313	0.12	0.168	0.35	<0.001	-0.16	0.081	0.07	0.428
AST (IU/L)	-0.09	0.326	0.07	0.406	0.23	0.010	-0.14	0.114	0.04	0.658
γGT (IU/L)	-0.19	0.028	-0.14	0.124	0.11	0.236	-0.21	0.017	-0.16	0.069
ALP (IU/L)	-0.14	0.126	-0.21	0.020	-0.06	0.509	-0.15	0.094	-0.20	0.022
Billirubin (mg/dL)	0.14	0.125	-0.01	0.896	0.27	0.002	0.08	0.391	-0.05	0.562
Hemoglobin (g/L)	0.07	0.456	0.27	0.002	0.23	0.009	0.03	0.759	0.23	0.010
Ferritin	0.25	0.006	0.12	0.192	NA	NA	0.08	0.389	-0.03	0.716
Albumin (g/L)	0.09	0.309	0.20	0.029	0.14	0.126	0.07	0.466	0.18	0.043
INR	0.07	0.420	0.01	0.865	0.15	0.092	0.05	0.555	-0.01	0.918
Liver hepcidin mRNA	NA	NA	0.08	0.396	0.25	0.006	NA	NA	0.05	0.585
Serum hepcidin	0.08	0.396	NA	NA	0.12	0.192	0.06	0.505	NA	NA

NA: not applicable.

As expected by the fact that iron overload is a stimulus for hepcidin transcriptional up-regulation, hepatic hepcidin mRNA expression correlated positively with ferritin (Table 2; P = 0.006). In contrast, no associations were found between serum hepcidin and ferritin or between liver hepcidin mRNA and serum hepcidin (Table 2). Liver hepcidin mRNA and hepcidin mRNA/log ferritin ratio correlated negatively with γ-GT (P<0.03 and P<0.02, respectively; Table 2). Serum hepcidin and serum hepcidin/log ferritin ratio associated positively with hemoglobin (P = 0.002 and P = 0.01, respectively) and albumin (P<0.03 and P<0.05, respectively) and negatively with ALP (P = 0.02 for both). Neither serum hepcidin nor liver hepcidin mRNA was significantly associated with inflammation grading or staging, though there was a tendency for increased serum hepcidin concentration in patients with severe fibrosis/cirrhosis compared to patients with none/mild/moderate fibrosis (P = 0.067; Table 3). In contrast, ferritin levels were significantly higher in moderate/severe inflammatory activity [162 (180) ng/ml] compared to minimal/mild activity [107 (114); P = 0.007] (Table 3).

**Table 3:** Correlations between liver histology, hepcidin and ferritin in all patients.

Parameters	Liver hepcidin mRNA	*P*	Serum hepcidin (ng/ml)	*P*	Ferritin (ng/ml)	*P*
**Inflammation**		0.716		0.176		0.007
*Minimal/mild*	0.745 (1.05)		40.6 (115.6)		107 (114)	
*Moderate/severe*	0.727 (1.45)		13.5 (60.4)		162 (180)	
**Fibrosis**		0.495		0.067		0.847
*None/mild/moderate*	0.744 (1.23)		25.6 (97.6)		120 (141)	
*Severe/cirrhosis*	0.839 (1.31)		56.2 (120.6)		106 (106)	

Values are expressed as the median and IQR.

That low serum hepcidin is a true characteristic of patients with PBC/PSC and AIH was confirmed by multivariate analysis. More specifically, predictor factors for serum hepcidin levels were assessed through a stepwise linear regression model in the total group of the patients (n = 126). In this model, serum hepcidin was the dependent variable, while the presence of AIH or PBC/PSC along with factors found to be significant in the univariate analysis (age, gender, hemoglobin, albumin, ALP levels and fibrosis stage) were included as independent variables. Ferritin levels were also included in order to assess their impact as a confounding factor. By using this analysis (Table 4), the type of liver disease (AIH or PBC/PSC) was revealed as the strongest predictor factor of serum hepcidin levels (P = 0.02). The remaining variables found predictive on univariate analysis added no further improvement in prediction.

**Table 4:** Predictors of serum hepcidin levels, considering presence of AIH or PBC/PSC as a comprehensive binary (present versus absent) covariate.

	β-coefficient	*P*
Age	-0.119	0.181
Gender	-0.114	0.203
Hemoglobin	0.081	0.362
Albumin	0.138	0.120
ALP	-0.173	0.055
Fibrosis stage	0.116	0.195
Ferritin levels	-0.067	0.451
Presence of AIH or PBC/PSC	-15.211	0.020

In the subgroup of patients with AIH or PBC/PSC (n = 50), liver hepcidin mRNA was significantly lower in females [0.43 (1.1)] compared to males [1.29 (0.98); p = 0.036] and associated positively with ferritin levels (P = 0.035). The analysis in patients with AIH (n = 16) also showed that females are characterized by decreased hepatic hepcidin mRNA expression [0.3 (0.44) versus 1.56 (0.87); P = 0.004] and, in the same group, serum hepcidin levels associated positively with INR (P = 0.003). In the patients with PBC/PSC (n = 34), liver hepcidin mRNA correlated positively with ferritin levels (P = 0.022). Regarding the fibrosis stage, serum hepcidin levels and serum hepcidin/log ferritin ratio were significantly increased in patients with severe fibrosis/cirrhosis compared to patients with none/mild/moderate fibrosis either in the combined group of AIH and PBC/PSC patients [16.6 (19.1) versus 8.4 (7.2); P = 0.017 and 9.7 (11.8) versus 4 (3.8); P = 0.018, respectively] or in AIH patients separately [12.9 (71.8) versus 8.3 (5.2); P = 0.027 and 6.2 (41.05) versus 3.4 (2.19); P = 0.038, respectively], while no association was revealed in PBC/PSC patients.

### Serum hepcidin levels in patients with autoimmune liver diseases during the course of treatment

**Fig 2:**
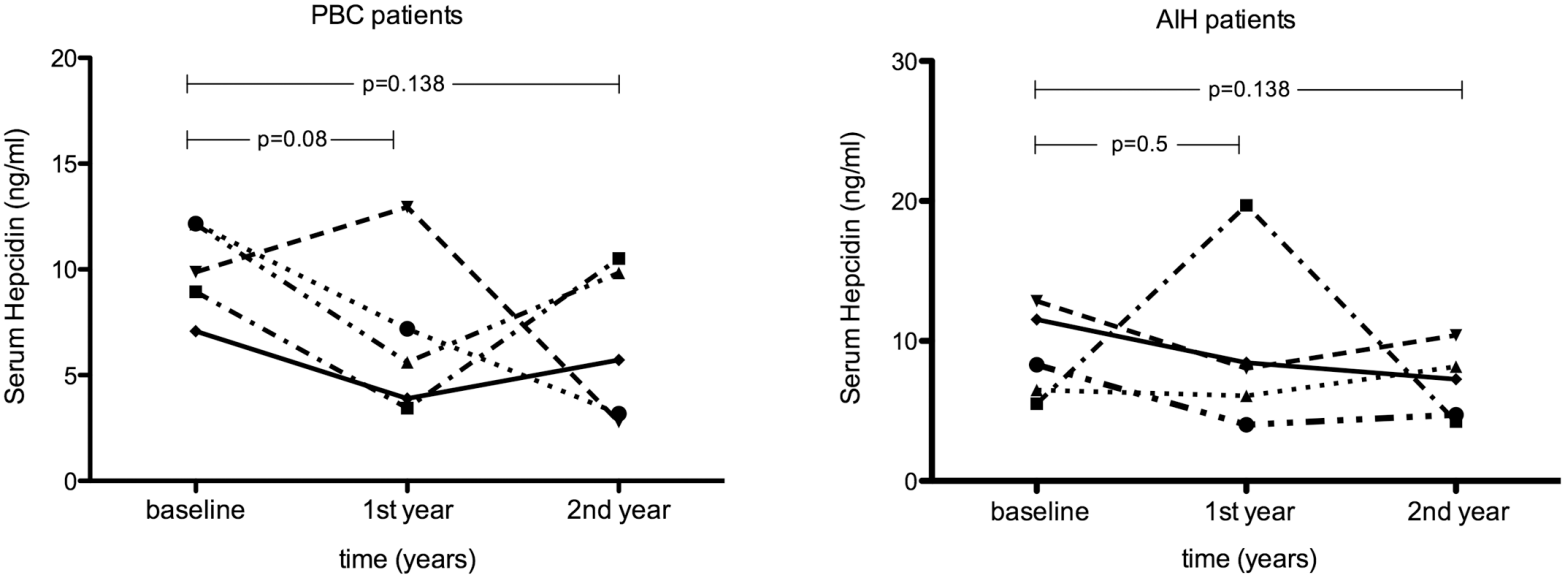
Serum hepcidin levels in patients with autoimmune liver diseases during treatment. Serum hepcidin levels in patients with PBC (*n* = 5, left panel) and AIH (*n* = 5, right panel) during a two-year treatment. *P* values calculated using Wilcoxon Signed-Rank test.

To gain an insight of the etiology behind low serum hepcidin concentration in patients with autoimmune liver diseases, we investigated the sera of ten non-cirrhotic patients in the course of their subsequent two-year conventional treatment. Five patients with PBC having low serum hepcidin at the time of first biopsy [9.9 (4.2)] were re-analysed after one and two years of treatment. As shown in Fig 2 (left panel), no significant alterations of serum hepcidin concentration were observed compared to baseline levels [1^st^ year, 5.6 (6.4); P = 0.08 and 2^nd^ year, 5.7 (7.2); P = 0.138, respectively). Similarly, in the group of five AIH patients (Fig 2, right panel) serum hepcidin levels were almost stable without significant differences during the two-year period of treatment (P = 0.5 for the 1^st^ year and P = 0.138 for the 2^nd^ year.

## Discussion

In the present study, we assessed liver hepcidin mRNA expression and serum hepcidin levels as well as their corresponding ratios to ferritin in patients with non-iron related liver disorders including for the first time patients with autoimmune liver diseases. The most important point arising from the present investigation is that serum hepcidin concentrations alone, as well as their corresponding ratios to serum ferritin, were extremely reduced in patients with AIH or PBC/PSC compared to any of the study groups and these levels remained low despite adequate treatment during a 2-years follow-up period. It should be stressed that the serum hepcidin levels observed in the AIH and PBC/PSC patients are not lower only when compared to the other patients of our cohort but they are also lower when compared to the “normal” concentrations of serum hepcidin as determined in healthy controls. Although absolute values given by in-house ELISA methods are not directly comparable (because of the use of different antibodies and peptide preparations), the values obtained for our healthy controls, median: 75 and 41 ng/ml for men and women, respectively, lie between those reported in the literature by previous bigger studies with healthy volunteers or general population, e.g. median: 112 (men) and 65 (women) ng/ml [42], median: 7.8 nM (21.8 ng/ml; men) and 6.5 nM (18.2 ng/ml; women) [24] and mean: 6.8 nM (19 ng/ml; men) and 3.8 nM (10.6 ng/ml; women) [43]. Of course, these differences in “normal” values may also be attributed to the different genetic backgrounds of the populations used in these studies.

In addition to the novel finding of low serum hepcidin in patients with autoimmune hepatitis, our study provides several interesting pieces of data that complement or confirm those from previously published works. Both liver hepcidin mRNA and serum hepcidin were significantly lower in female patients of our cohort, which, at least partially, can be due to the lower iron stores in the same patients as depicted by the strong negative correlation of ferritin with the female gender. This has been previously reported, for serum hepcidin, in patients with chronic hepatitis C [44] and in healthy volunteers or the general population, as described above [24, 42, 43]. Serum hepcidin and its ratio to ferritin negatively correlated with age in all patients. This finding could be potentially explained by the high prevalence of anemia in the elderly, though further studies are required in order to elucidate the effect of aging on hepcidin regulation [45]. The AIH and PBC/PSC groups, which showed the lowest serum hepcidin concentration, have a high prevalence of women and older patients with the older mean age noticed in the PBC/PSC group. These group compositions are normal, as both primary biliary and autoimmune hepatitis are characterized by female predominance (8:1 and 4–5:1, respectively). Besides, primary biliary cirrhosis is a liver disease affecting mainly middle-aged women. On the other hand, chronic viral hepatitis and particularly chronic hepatitis B affect predominantly males. As both age and female gender are associated with low serum hepcidin, one might possibly argue that low serum hepcidin is not related to the type of the disease but rather to the particular composition of the disease group. We believe that this is highly unlikely for at least two reasons. First, stepwise linear regression analysis clearly reveals the type of liver disease (AIH or PBC/PSC) as the only statistically significant predictor of serum hepcidin levels, irrespectively of age and gender (Table 4). Second, when we restricted the statistical analysis only in female patients (n = 74) in order to investigate the impact of gender, the results were identical to those noticed in the whole group of patients (n = 126), with significantly lower levels of serum hepcidin and serum hepcidin/log ferritin ratio in autoimmune liver diseases groups (p<0.001; S1 Table). Besides, in the subgroup of female patients with chronic liver diseases, ANOVA analysis showed that there was no significant difference regarding age among disease groups (p = 0.061). Nevertheless, as the relatively small size of our cohort and the imperfect matching in the different disease groups may, somehow, bias the results of our study, our findings need to be confirmed by follow-up studies involving more patients and better-matched subgroups regarding age and gender.

Hepatic hepcidin mRNA expression correlated with serum ferritin depicting the direct effect of iron concentration on the transcriptional activation of hepcidin. Serum hepcidin positively correlated with hematological parameters (albumin, hemoglobin) supporting a possible role of reduced erythropoiesis and anemia on circulating hepcidin levels whereas liver hepcidin mRNA and serum hepcidin correlated negatively with cholestatic markers in agreement with previous findings [27, 46]. Liver hepcidin mRNA, serum hepcidin levels and their ratios to ferritin were lower in HCV patients compared to HBV but the differences did not reach in all cases statistical significance as reported previously for the hepcidin/serum ferritin ratio [27, 28] and more work is needed to definitely confirm diminished hepcidin expression levels in the HCV-infected liver.

The HCV patient group as well as the AIH group had the highest percentage of patients with moderate to severe inflammation (43–44%) while the HBV group the highest proportion (43.5%) of severe fibrosis as judged by the histological analysis. Although, no significant association could be shown between liver hepcidin mRNA or serum hepcidin and inflammation severity or fibrosis, patients with higher grading score for inflammation had lower serum hepcidin while patients with severe fibrosis had a tendency for higher serum hepcidin. On the other hand, inflammation and fibrosis did not have an effect on liver hepcidin mRNA. These findings are unexpected as they contrast with a previous study showing negative correlation between fibrosis status and liver hepcidin mRNA or urinary hepcidin [47]. However, this particular study analyzed patients with end-stage disease (being operated on for liver carcinoma or receiving transplants for cirrhosis) in contrast with our analysis of patients at the time of first diagnosis and with, conceivably, early stage liver disease. This, plus the fact that our study includes a relatively small number of patients with advanced liver inflammation and/or fibrosis could explain the lack of significant association between hepcidin levels (either at mRNA or circulating peptide levels) and liver inflammation or cirrhosis. Another finding in support of this lack of correlation is that serum hepcidin levels as well as the serum hepcidin:ferritin ratio was similar between the AIH and PBC/PSC groups, despite the differences in inflammation severity among them (43.8% with moderate/severe inflammation in AIH vs. 23.5% in PBC/PSC group). However, the positive correlation between serum hepcidin and albumin indicates that some relationship may exist between hepatic damage and hepcidin synthesis as also supported by the previous study [47]. The design of our study does not allow validation of this hypothesis, because, as already mentioned, only six cirrhotic patients were included in the study, all of them had compensated cirrhosis with preserved liver function and, as a result, multivariate analysis excluded albumin as a predictor factor of serum hepcidin levels in our set of patients (Table 4). Therefore, future studies including patients with decompensated liver disease are needed to clarify this issue.

The lower serum hepcidin levels in PBC/PSC and AIH are probably not the result of inhibition of liver hepcidin mRNA expression levels, as these levels did not differ significantly among the study groups, with a single exception (lower hepcidin mRNA in AIH when compared to HBV). This may be surprising but it is in agreement with the fact that hepcidin gene expression levels in the liver (the principal site of hepcidin production) do not correlate with levels of hepcidin mature peptide in the serum of our patients and it is not without precedent. Previous studies that have determined liver hepcidin mRNA as well as urinary or serum hepcidin in patients reported different results concerning the correlation between these values. A moderate correlation was observed between urinary hepcidin concentrations and hepatic hepcidin mRNA values in patients with liver cancer or cirrhosis [47] but not in patients with dysmetabolic hepatic iron overload [48]. In studies analyzing patients with chronic hepatitis C, liver hepcidin mRNA has been both correlated [49] and not correlated [50] with serum hepcidin, while two recent studies involving patients with different hepatic disorders demonstrated a correlation between the amount of hepcidin in serum and its liver transcript [27, 51].

There are several possible explanations for theses inconsistencies. First, changes in hepcidin mRNA expression may be subtler than changes in secreted peptide amounts and, therefore, more difficult to become statistically significant with a relatively small group of subjects or due to restrictions in the accuracy and the detection limits of the measurement methods. In this respect, it should be noted that AIH patients, the smallest group in our study, show both the lowest serum hepcidin (compared to all other non-autoimmune groups) and the lowest liver hepcidin mRNA, but the latter differs significantly only when compared to the HBV group. Second, extrahepatic sources can also contribute to the secretion of hepcidin [52], but their contribution is generally small and very hard to define, especially in a patient population. Finally, several post-transcriptional processes such as translation, intracellular trafficking, processing, secretion, binding to ferroportin and internalization are also involved in determining the final and functional levels of hepcidin in the circulation. We have recently reported that in cultured human hepatoma cells hepcidin secretion can be regulated by post-transcriptional mechanisms and is inhibited, for example, by low oxygen levels [53]. Thus, it is possible that factors related to the etiology of PBC/PSC and AIH affect the post-transcriptional processing of the hepcidin pre-pro-peptide resulting in low levels of mature hepcidin secretion without corresponding changes in the levels of its hepatic mRNA.

Although AIH and PBC/PSC are complex autoimmune liver diseases that differ in autoimmune injury and clinical phenotype, the diminished serum hepcidin levels found in our study seem to be a common characteristic, which may either might be associated with the pathogenicity or be a specific consequence of liver autoimmunity. The answer to these questions clearly necessitates much additional investigation but one speculation that could explain our data is the involvement of hepcidin in autoimmunity. Despite being critical for iron homeostasis, hepcidin is an acute phase peptide of the innate immunity with antimicrobial properties. Both roles of hepcidin are tightly related to each other, since iron homeostasis affects immune defense and vice versa. Hepatocyte production of hepcidin is responsive to body iron stores, while as a member of the β-defensin family [54] hepcidin limits the availability of iron for microorganism growth and survival [55]. Autoimmune diseases are often induced or triggered by infectious agents. Under physiological conditions the interplay between the innate and the adaptive immune system leads to efficient suppression of the pathogenic microorganism within few days. A defect in the adaptive immune system or a less specific activation of the innate immune response, in this case due to low hepcidin levels, could break peripheral tolerance or ignorance and promote autoimmune disease [56]. Resembling the antimicrobial spectrum of human β-defensin-1 [1], hepcidin is chemotactic for T cells, dendritic cells, monocytes and mast cells. Down-regulation of serum hepcidin levels could be the cause of an imperfect interplay between the innate and the adaptive immune system in patients with autoimmune liver diseases. In this hypothesis, the low levels of circulating hepcidin could be a causative factor of liver autoimmunity, similar to the decreased expression of defensin DEFB1 that leads to impaired antimicrobial activity in patients with Crohn's disease [57]. This hypothesis can only be tested by future, well-designed, bigger and prospective clinical studies.

In conclusion, parallel determination of hepcidin expression levels in liver biopsies and sera of patients with different hepatic disorders has revealed that serum hepcidin concentrations and their corresponding ratios to ferritin are dramatically low in patients with autoimmune liver diseases in comparison to other liver diseases. This striking and novel finding necessitates further studies to evaluate the role of serum hepcidin as a sensitive biomarker for liver autoimmunity and its pathogenic involvement along with the underlying molecular mechanisms.

## Supporting Information

S1 Table Results of statistical analysis of the female patient population data (DOCX)
