# Supplementary material for: Low Serum Hepcidin in Patients with Autoimmune Liver Diseases
Source: PLoS One. 2015 Aug 13;10(8):e0135486. doi: 10.1371/journal.pone.0135486 (PMC4535884; doi:10.1371/journal.pone.0135486)
Supplement: S1 Table — (DOCX) [file pone.0135486.s001.docx]

**Supplementary Table 1.** Results of statistical analysis of the female patient population data

| **Variables** | **Total**  **(n=74)** | **HCV**  **(n=11)** | **HBV**  **(n=8)** | **AIH**  **(n=12)** | **PBC/PSC**  **(n=27)** | **NAFLD**  **(n=16)** | ***P*-value** |
| --- | --- | --- | --- | --- | --- | --- | --- |
| Age  (years) | 51.4±14.4 | 44±11.1 | 49.3±16.7 | 55.2±11.9 | 57±10.9 | 48.8±17.9 | 0.061 |
| Ferritin  (ng/ml) | 84 (108) | 46 (91) | 83 (158) | 83 (157) | 66 (106) | 122 (81) | 0.558 |
| Liver hepcidin  mRNA | 0.58 (1.13) | 0.71 (1.18) | 0.99 (2.56) | 0.29 (0.44) | 0.60 (1.01) | 0.66 (1.07) | 0.206 |
| Hepcidin mRNA /  log ferritin | 0.29 (0.60) | 0.44 (0.72) | 0.45 (1.32) | 0.16 (0.25) | 0.27 (0.63) | 0.35 (0.58) | 0.193 |
| Serum hepcidin  (ng/ml) | 20 (94) | 58 (221) | 154 (154) | 8 (7) | 9 (13) | 69 (97) | <0.001 |
| Serum hepcidin /  log ferritin | 8.9 (46.1) | 32.3 (131.7) | 85 (125.3) | 4.3 (3.3) | 4.5 (5.4) | 34 (47.9) | <0.001 |
